# Gut microbiota and sepsis: bidirectional Mendelian study and mediation analysis

**DOI:** 10.3389/fimmu.2023.1234924

**Published:** 2023-08-17

**Authors:** Zhi Zhang, Lin Cheng, Dong Ning

**Affiliations:** ^1^ Department of Health Management, The First Affiliated Hospital of Hunan University of Traditional Chinese Medicine, Changsha, Hunan, China; ^2^ Regenerative Medicine Institute, School of Medicine, National University of Ireland (NUI), Galway, Ireland; ^3^ Discipline of Physiology, Human Biology Building, School of Medicine, National University of Ireland (NUI), Galway, Ireland

**Keywords:** C-reactive protein, gut microbiota, mediator, Mendelian randomization, sepsis

## Abstract

**Background:**

There is a growing body of evidence that suggests a connection between the composition of gut microbiota and sepsis. However, more research is needed to better understand the causal relationship between the two. To gain a deeper insight into the association between gut microbiota, C-reactive protein (CRP), and sepsis, we conducted several Mendelian randomization (MR) analyses.

**Methods:**

In this study, publicly available genome-wide association study (GWAS) summary statistics were examined to determine the correlation between gut microbiota and sepsis, including various sepsis subgroups (such as under 75, 28-day death, Critical Care Units (ICU), 28-day death in ICU). Initially, two-sample and reverse Mendelian randomization (MR) analyses were conducted to identify causality between gut microbiota and sepsis. Subsequently, multivariable and two-step MR analyses revealed that the relationship between microbiota and sepsis was mediated by CRP. The robustness of the findings was confirmed through several sensitivity analyses.

**Findings:**

In our study, we revealed positive correlations between 24 taxa and different sepsis outcomes, while 30 taxa demonstrated negative correlations with sepsis outcomes. Following the correction for multiple testing, we found that the Phylum Lentisphaerae (OR: 0.932, *p* = 2.64E-03), class Lentisphaeria, and order Victivallales (OR: 0.927, *p* = 1.42E-03) displayed a negative relationship with sepsis risk. In contrast, Phylum Tenericutes and class Mollicutes (OR: 1.274, *p* = 2.89E-03) were positively related to sepsis risk and death within 28 days. It is notable that Phylum Tenericutes and class Mollicutes (OR: 1.108, *p* = 1.72E-03) also indicated a positive relationship with sepsis risk in individuals under 75. From our analysis, it was shown that C-reactive protein (CRP) mediated 32.16% of the causal pathway from Phylum Tenericutes and class Mollicutes to sepsis for individuals under 75. Additionally, CRP was found to mediate 31.53% of the effect of the genus Gordonibacter on sepsis. Despite these findings, our reverse analysis did not indicate any influence of sepsis on the gut microbiota and CRP levels.

**Conclusion:**

The study showcased the connection between gut microbiota, CRP, and sepsis, which sheds new light on the potential role of CRP as a mediator in facilitating the impact of gut microbiota on sepsis.

## 1 Background

Sepsis is a complex syndrome characterized by an unbalanced immune response to various infections (1), which can lead to malfunctioning of multiple organ systems such as the cardiopulmonary, renal, and digestive systems (2). According to epidemiological studies, sepsis rates of prevalence and mortality range from 25% to 30% in hospitals (3). Despite our growing understanding of the biological mechanisms behind sepsis, current treatments have proven ineffective in correcting the dysregulated immunity in patients (4), making it essential to develop targeted prevention and treatment strategies.

The gut microbiome has been found to contribute to the severity of sepsis and prognosis of treatment (5). Preclinical studies have shown that gut microbiota plays a pivotal role in the immune response to systemic inflammation and that disruption of this symbiosis increases susceptibility to sepsis (6). Additionally, the use of omic technologies to analyze the gut microbiota has confirmed the alteration of composition related to septic dysfunction across organs (7).

Although probiotic supplementation has reported some positive effects (8–10), their efficacy and safety remain a subject of controversy (11, 12). Therefore, more research is necessary to identify the specificity and safety of probiotic supplements.

In addition to being a biomarker of acute-phase inflammation, CRP has a role in defending against infections as it can bind to cells and some bacteria, triggering the complement system and helping to remove dead cells (13, 14). However, prospective studies have also revealed that elevated CRP levels correlate with a higher risk of infections in adults (15).

Mendelian randomization (MR) involves using genetic variants to construct instrumental variables of exposure and estimate the causal association between exposure and outcome (16). As the random distribution of alleles is not affected by common confounding factors, a causal relationship is generally considered to be reliable (17). However, in previous studies, we were unable to find any MR studies examining the relationship between gut microbiota, sepsis, and their association with CRP. Therefore, we conducted multiple MR analyses based on genome-wide association study (GWAS) summary statistics to evaluate the causal association among gut microbiota, CRP, and sepsis.

## 2 Method

### 2.1 Study design

**Figure 1:**
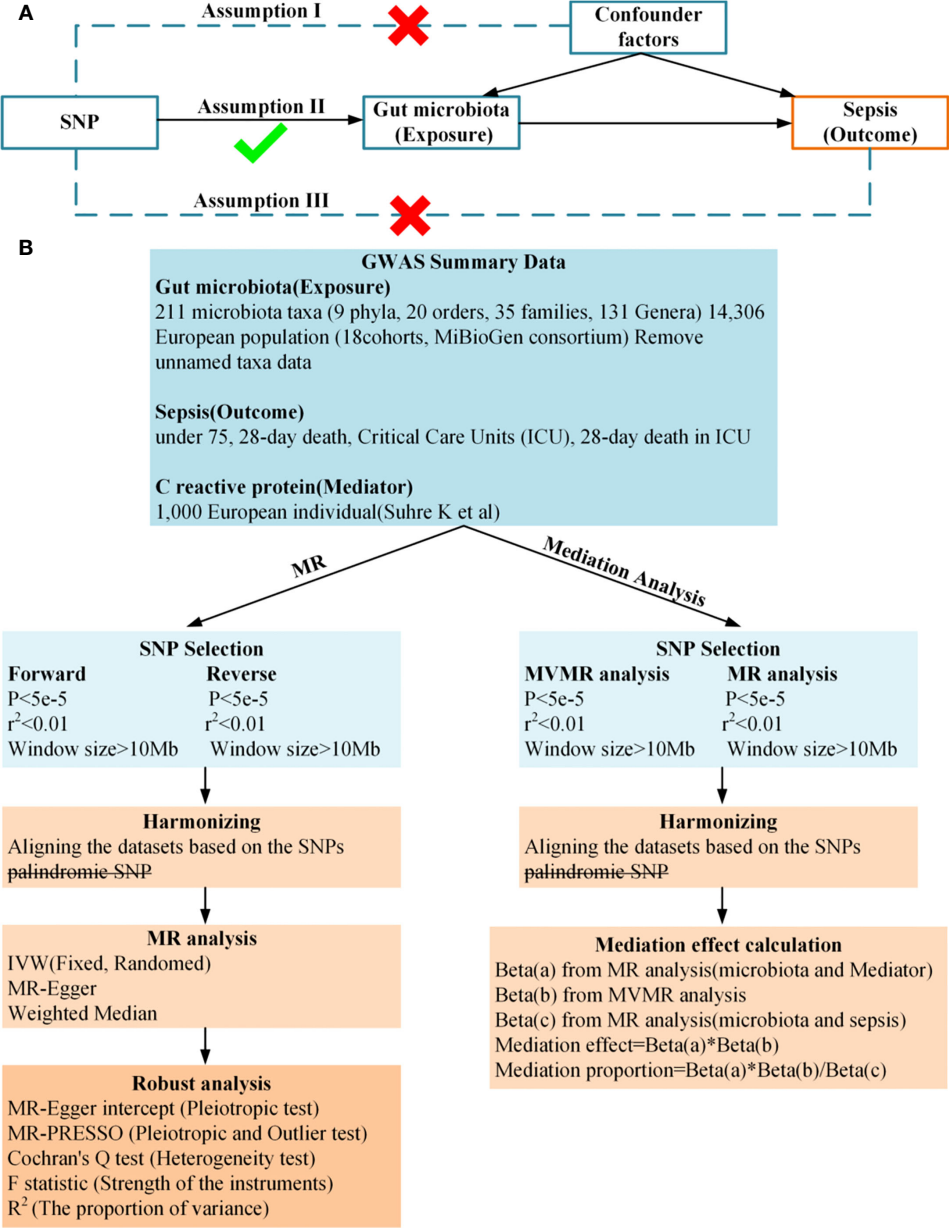
**(A)**, Principles of Mendelian Randomization: I) Independence: The genetic variants utilized in the analysis are not associated with any confounders that could potentially influence the relationship between the exposure and the outcome. II) Relevance: The genetic variants selected as instrumental variables have a strong association with the exposure. III) Exclusion Restriction: The genetic variants influence the outcome solely through their effect on the exposure, and not through any alternative pathways; **(B)**, Flowchart of Bidirectional Two-Sample Mendelian Randomization and mediation Analysis.

In this study, we conducted a two-sample and bidirectional Mendelian randomization (MR) to examine the causal relationship between gut microbiota and sepsis. We then used a two-step and multivariable MR approach to identify the mediation effect of CRP on the relationship between gut microbiota and sepsis. A summary of the study design is illustrated in **Figure 1**. Study used publicly available summary statistics for gut microbiota, C-reactive protein (CRP), and sepsis from previously published studies or consortiums. All of these studies were approved by their respective institutional review boards (IRBs), and hence, there was no need to re-apply for approval by the IRB.

### 2.2 Data sources

The gut microbiota data used in this study were sourced from the MiBioGen consortium (18). This consortium has curated and analyzed genome-wide genotypes and 16S fecal microbiome data from 18,340 individuals across 24 cohorts, which includes 14,306 European individuals from 18 cohorts. The consortium performed adjustments for age, sex, genetic principal components, technical covariates such as stool DNA isolation methods, 16S domain to reduce heterogeneity among the cohorts. However, the study did not account for other potential confounders like diet, medication use, and lifestyle factors, as this information was not consistently available for all cohorts (**Supplementary Table 1**).

C-reactive protein was derived from 1,000 individuals in the population-based KORA (Cooperative Health Research in the Region of Augsburg) study (19). The study used a highly multiplexed, aptamer-based, affinity proteomics platform (SOMAscan) to quantify levels of 1,124 proteins in blood plasma samples.

The sepsis data and sepsis subgroups (under 75, 28-day death, Critical Care Units (ICU), 28-day death in ICU) was collected from the IEU Open GWAS with summary-level data obtained from the UK Biobank which included 11643,11568,1896,1380, and 347 sepsis cases and 474841,451301,484588, 429985, 431018 controls respectively. The study use Regenie v2.2.4 to analyze GWAS data, and adjusted for age, sex, chip, and the first 10 Principal Component Analysis (https://gwas.mrcieu.ac.uk/datasets/ieu-b-4980/).

### 2.3 SNP selection

We utilized MR analysis to investigate potential causal relationships between gut microbiota and sepsis, using genetic variants as instrumental variables (IVs). The validity of an MR analysis is contingent upon three key assumptions: (1) IVs are not associated with any confounding variables; (2) IVs are strongly associated with the exposure; and (3) IVs influence the outcome solely through the exposure (20).

Initially, we selected single nucleotide polymorphisms (SNPs) from the genome-wide association study (GWAS) summary data for exposures that exhibited a genome-wide significant association (p < 5×10^−8^) with the traits as IVs. In instances where the number of IVs was limited, we relaxed the significance threshold to 5×10^−5^ to prevent inaccurate results due to insufficient SNPs. The selection of other SNPs followed the same threshold. Subsequently, we employed linkage disequilibrium clumping to exclude certain undesirable SNPs (r^2^ < 0.01, window size > 10,000 kb) (21). Finally, we harmonized the exposure and outcome datasets and eliminated palindromic SNPs with allele frequencies close to 0.5. All the selected SNPS are placed in the **Supplementary Table 2.**

We ensured the strength of genetic instruments for exposures by calculating the F statistic using the formula:*F* = (n - k − 1)/k×(R^2^/1− R^2^) (22), where R^2^ represents the cumulative explained variance in the selected SNPs, N is the sample size, and k is the number of SNPs in the analysis. An F statistic greater than 10 indicates sufficient strength to avoid the issue of weak instrument bias in the two-sample model (23).

### 2.4 Statistical analysis

We conducted bidirectional two-sample MR analyses to assess the relationship between gut microbiota and sepsis. Our primary analysis employed an inverse variance-weighted (IVW) meta-analysis approach, which is a robust method for MR analysis (17). We also performed secondary analyses using the weighted median (24), and MR-Egger regression approaches. We evaluated the potential impact of directional pleiotropy by testing the intercept value of the MR-Egger regression (25). We used Cochran’s Q test to assess heterogeneity (26). In cases of heterogeneity, we opted for a random-effects IVW for the primary analysis. At each feature level (phylum=9, class=15, order=19, family=30, and genus=117), according to previous reports (27), we used a multiple-testing significance threshold of *p* < 0.05/n (where n represents the effective number of independent bacterial taxa at the corresponding taxonomic level).

In mediation terms, the total effect of an exposure on the outcome is estimated by univariable MR. Multivariable MR (MVMR) and two-step MR is used to decompose direct and indirect effects. The first step is to evaluate the effect of exposure on the mediator with univariable MR. The second step estimating the effect of the mediator on each outcome was carried out with MVMR. For this second step, MVMR has not been used in previous literature (28, 29), and a univariable MR has been proposed for calculating the mediator’s effect. However, in the case of MVMR, the mechanism of the mediator’s effect on the outcome can be ensured to be independent of the effect of the exposure (30). Furthermore, it exerts a direct effect on exposure. The indirect effect is estimated by multiplying the two-step (MR) estimates. Stepwise regression was used to select exposures and mediators with true effects (31).

## 3 Result

### 3.1 Two-Sample and bidirectional MR analysis of gut microbiota and sepsis, sepsis subgroups risk

**Figure 2:**
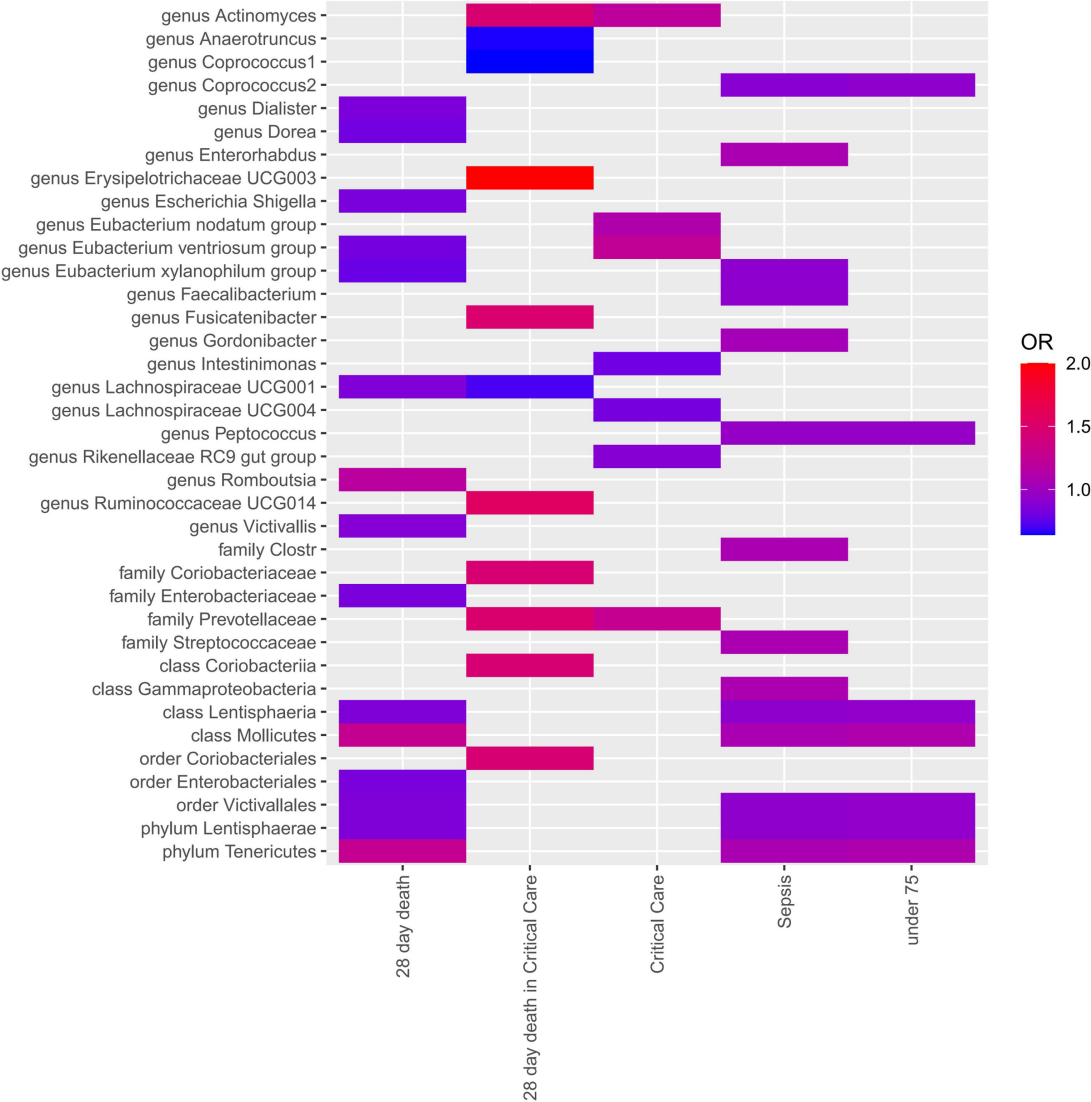
Each cell in the heatmap corresponds to a specific micrbiota taxa-sepsis pair. The color of the cell indicates the OR value associated with that pair, with a color scale used to differentiate between positive, negative, and zero beta values.

Four MR approaches were utilized to investigate the association between gut microbiota and sepsis (**Figure 2** and **Supplementary Table 3**). Positive associations were observed for the genera Actinomyces, Enterorhabdus, Gordonibacter, and Ruminococcaceae UCG014, and the families Coriobacteriaceae and Prevotellaceae, with various outcomes. For example, the genus Actinomyces was associated with an increased likelihood of critical care units (OR = 1.21, *p* = 2.58E-02) and 28-day death in critical care units (OR = 1.46, *p* = 2.58E-02). The genus Fusicatenibacter demonstrated a strong positive association with 28-day death in critical care units(OR = 1.49, *p* = 3.90E-02). In contrast, several taxa showed negative associations with sepsis outcomes. For instance, the genera Anaerotruncus, Coprococcus1, Coprococcus2, Dialister, Dorea, Eubacterium ventriosum group, Eubacterium xylanophilum group, Faecalibacterium, Intestinimonas, Lachnospiraceae UCG001, Lachnospiraceae UCG004, and Peptococcus, and the family Enterobacteriaceae, all demonstrated negative associations with various outcomes.

Notably, the genus Erysipelotrichaceae UCG003 displayed a particularly strong positive association with 28-day death in ICU (OR = 4.97, *p* = 2.43E-02), suggesting a potential role in severe sepsis outcomes. The genus Eubacterium xylanophilum group showed negative associations with both 28-day death (OR = 0.78, *p* = 3.26E-03) and sepsis (OR = 0.92, *p* = 1.68E-02), suggesting a protective role.

Multiple-testing correction was taken into account by setting significance thresholds as follows: phylum *p* = 5.56×10^-3^ (0.05/9), class *p* = 3.13×10^-3^ (0.05/16), order *p* = 2.63 × 10^-3^ (0.05/19), family *p* = 1.67 × 10^-3^ (0.05/30), genus *p* = 4.27 × 10^-4^ (0.05/117). As the SNPs within a class might overlap with those in a related phylum and other subcategories, the MR results would remain similar if a class was considered a subcategory of a phylum or another subcategory.

Based on the results of IVW fixed-effects analyses **Table 1**, phylum Lentisphaerae (OR = 0.932, 95% CI = 0.89-0.98, *p* = 2.64E-03), class Lentisphaeria and order Victivallales (OR = 0.927, 95% CI = 0.88-0.97, *p* = 1.42E-03) were negatively associated with the risk of Sepsis. Conversely, phylum Tenericutes and class Mollicutes (OR = 1.274, 95% CI = 1.09-1.49, *p* = 2.89E-03) were positively correlated with the risk of Sepsis (28 day death). Interestingly, Phylum Tenericutes and class Mollicutes (OR = 1.108, 95% CI = 1.04-1.18, *p* = 1.72E-03) were positively correlated with the risk of Sepsis (under 75 years) as well. No effect of sepsis on gut microbiota was found in the reverse analysis (**Supplementary Table 4**).

**Table 1:** MR result of gut microbiota on sepsis.

Exposure	Methods	Number of SNPs	OR	95%CI	*p* val	Cochran’s Q statistic (*p* val)	Egger intercept(*p* val)	F
Sepsis(Outcome)								
phylum Lentisphaerae	IVW-FE	41	0.932	0.89-0.98	**2.64E-03**	0.31	0.979	19.67
	IVW-RE		0.932	0.89-0.98	**4.11E-03**			
	MR Egger		0.93	0.78-1.11	0.429			
	WM		0.969	0.91-1.04	0.347			
class Lentisphaeria order Victivallales	IVW-FE	40	0.927	0.88-0.97	**1.42E-03**	0.663	0.982	19.56
	IVW-RE		0.927	0.88-0.97	**1.42E-03**			
	MR Egger		0.929	0.78-1.1	0.406			
	WM		0.958	0.89-1.03	0.234			
Sepsis (28 day death)(Outcome)								
phylum Tenericutes class Mollicutes	IVW-FE	46	1.274	1.09-1.49	**2.89E-03**	0.691	0.489	19.45
	IVW-RE		1.274	1.09-1.49	**2.89E-03**			
	MR Egger		1.099	0.7-1.71	0.68			
	WM		1.288	1.02-1.63	0.034			
Sepsis(under 75)(Outcome)								
phylum Tenericutes class Mollicutes	IVW-FE	46	1.108	1.04-1.18	**1.72E-03**	0.204	0.27	19.5
	IVW-RE		1.108	1.03-1.19	**3.73E-03**			
	MR Egger		1	0.83-1.21	0.999			
	WM		1.105	1-1.22	0.047			

IVW-FE, Inverse variance weighted-Fixed model; IVW-RE, Inverse variance weighted-Random model; WM, weight median; F is the value of F statistics to examine the weak instrument bias; Significant p-values were bold after multiple-testing correction [phylum p = 5.56×10^-3^ (0.05/9), class p = 3.13×10^-3^ (0.05/16), order p = 2.63×10^-3^ (0.05/19), family p = 1.67×10^-3^ (0.05/30), genus p = 4.27×10^-4^ (0.05/117)].

For additional confirmation of the robustness of the results, several sensitivity tests were conducted (**Supplementary Table 4**). Most of the results were consistent in sensitivity analyses, though with wider confidence intervals. In addition, all results of Cochran’s Q test were above 0.05, signifying that there was no significant heterogeneity. The MR-PRESSO analysis also corroborated this, demonstrating no outlier of SNPs. Moreover, the MR-Egger intercept test and the global test p-values both revealed no statistically significant results, suggesting no presence of horizontal pleiotropy.

### 3.2 Gut microbiota and C-reactive protein level

Similarly, we conducted two-sample analyses to examine the relationship between gut microbiota and C-reactive protein (CRP). The IVW fixed-effects analyses **Table 2** showed that family Coriobacteriaceae, order Coriobacteriales, class Coriobacteriia had a negative correlation with CRP levels (Beta = -0.502 *p* = 0.046), However, Phylum Tenericutes, class Mollicutes, genus Dialister, genus Gordonibacter had a positive correlation with CRP levels, and WM analysis also obtained similar causal estimates.

**Table 2:** MR result of gut microbiota on CRP.

Exposure	Methods	Number of SNPs	Beta	Se	*P*val	Cochran’s Q statistic (*P*-value)	Egger intercept(*P*-value)	F
CRP(Outcome)								
family Coriobacteriaceae	IVW-FE	12	-0.502	0.252	**4.59E-02**	0.642	0.582	19.388
class Coriobacteriia	IVW-RE	12	-0.502	0.252	**4.59E-02**	0.642	0.582	19.388
order Coriobacteriales	MR Egger	12	-1.304	1.430	3.83E-01	0.642	0.582	19.388
	WM	12	-0.492	0.335	1.42E-01	0.642	0.582	19.388
class Mollicutes	IVW-FE	6	0.736	0.292	**1.17E-02**	0.878	0.760	18.688
phylum Tenericutes	IVW-RE	6	0.736	0.292	**1.17E-02**	0.878	0.760	18.688
	MR Egger	6	0.408	1.043	7.16E-01	0.878	0.760	18.688
	WM	6	0.819	0.370	**2.68E-02**	0.878	0.760	18.688
genus Dialister	IVW-FE	9	0.585	0.238	**1.41E-02**	0.267	0.463	18.297
	IVW-RE	9	0.585	0.266	**2.80E-02**	0.267	0.463	18.297
	MR Egger	9	1.523	1.238	2.58E-01	0.267	0.463	18.297
	WM	9	0.410	0.335	2.22E-01	0.267	0.463	18.297
genus Gordonibacter	IVW-FE	9	0.293	0.136	**3.10E-02**	0.927	0.822	18.667
	IVW-RE	9	0.293	0.136	**3.10E-02**	0.927	0.822	18.667
	MR Egger	9	0.162	0.574	7.86E-01	0.927	0.822	18.667
	WM	9	0.188	0.178	2.89E-01	0.927	0.822	18.667

IVW-FE, Inverse variance weighted-fixed model; IVW-RE, Inverse variance weighted-random model; WM, weight median; CRP, C reactive protein; F is the value of F statistics to examine the weak instrument bias.

Bold means that the p-value is less than 0.05.

A series of sensitivity analyses, including WM, Cochran’s Q test, MR-Egger regression, intercept test were conducted **Table 2**. These results were consistent in sensitivity analyses, though some with wider confidence intervals. Additionally, all *p* values from both the Cochran’s Q test and the MR-Egger intercept test were greater than 0.05, indicating the absence of heterogeneity and horizontal pleiotropy. The reverse analysis did not find any effect of CRP on gut microbiota (**Supplementary Table 5**).

### 3.3 C-reactive protein level and sepsis, sepsis subgroups

Initially, we conducted two-sample MR analyses (**Table 3**) to examine the effect of C-reactive protein levels on sepsis and its subgroups. The table presents the results, and the IVW fixed-effects analyses showed a positive correlation between CRP levels and Sepsis and Sepsis (under 75). No effect of sepsis on CRP was found in the reverse analysis (**Supplementary Table 6**). Furthermore, a series of sensitivity analyses validated the robustness of the findings.

**Table 3:** MR result of CRP on sepsis.

Exposure	Methods	Number of SNPs	OR	95%CI	*P*val	Cochran's Q statistic (*P*-value)	Egger intercept(*P*-value)	F
Sepsis(Outcome)								
CRP	IVW-FE	21	1.046	1.01-1.08	**0.006**	0.608	0.497	29.45
	IVW-RE	21	1.046	1.01-1.08	**0.006**			
	MR Egger	21	1.077	0.99-1.18	0.114			
	WM	21	1.036	0.99-1.09	0.135			
Sepsis (28 day death)(Outcome)								
CRP	IVW-FE	21	1.042	0.96-1.13	0.305	0.448	0.108	29.45
	IVW-RE	21	1.042	0.96-1.13	0.307			
	MR Egger	21	1.238	1-1.53	0.067			
	WM	21	1.073	0.96-1.2	0.215			
Sepsis (28 day death in Critical Care Units)(Outcome)								
CRP	IVW-FE	21	1.0823696	0.9	1.302	0.414	0.027	29.45
	IVW-RE	21	1.0823696	0.9	1.307			
	MR Egger	21	1.9199409	1.2	3.176			
	WM	21	1.137025	0.9	1.485			
Sepsis (under 75)(Outcome)								
CRP	IVW-FE	21	1.046	1.01-1.08	**0.005**	0.729	0.597	29.45
	IVW-RE	21	1.046	1.01-1.08	**0.005**			
	MR Egger	21	1.069	0.98-1.17	0.142			
	WM	21	1.054	1.01-1.1	**0.02**			

IVW-FE, Inverse variance weighted-fixed model; IVW-RE, Inverse variance weighted-random model; WM, weight median; CRP, C reactive protein; F is the value of F statistics to examine the weak instrument bias.

Bold means that the p-value is less than 0.05.

Secondly, we utilized MVMR (as shown in **Table 4**) to assess the independent impact of CRP on sepsis, which was independence of gut microbiota. The results indicate a significant positive association between CRP levels and a higher risk of sepsis as well as sepsis under 75 years old.

**Table 4:** MVMR result of gut microbiota and CRP on sepsis.

Exposure	Number of SNPs	OR	95%CI	*p*val
Sepsis (under 75)(Outcome)				
phylum Tenericutes/ class Mollicutes	23	1.1	0.93-1.3	0.26
CRP	23	1.05	1.01-1.08	**0.011**
Sepsis(Outcome)				
genus Gordonibacter	25	0.98	0.9-1.07	0.692
CRP	25	1.05	1.01-1.08	**0.011**

MVMR, Multivariable Mendelian randomization; IVW-FE, Inverse Variance Weighted-Fixed model; IVW-RE, Inverse Variance Weighted-Random model; CRP, C reactive protein; WM, Weight Median; Significant P-values were bold.

As shown in **Table 5**, the mediation analysis revealed that CRP plays a significant role (32.02% mediation effect) in the causal pathway from Phylum Tenericutes and class Mollicutes to sepsis (in individuals under 75 years old). And CRP mediate 31.53% effect of genus Gordonibacter on sepsis.

**Table 5:** Two-step Mendelian randomization.

Exposure	Mediation	Total effect (Beta)	A (Beta)	B (Beta)	Indirect effect (Beta)	Mediation effect/ Total effect
Sepsis (under 75)(Outcome)						
phylum tenericutes/ class Mollicutes	CRP	0.102	0.736	0.044	0.033	32.02%
Sepsis(Outcome)						
genus Gordonibacter	CRP	0.045	0.293	0.045	1.32%	31.53%

A, the effect of Exposure on Mediation; B, the effect of Mediation on Outcome is independent of the effect of the exposure.

## 4 Discussion

Over the past decade, numerous studies have confirmed the diverse biological functions of gut microbes, including aiding in food digestion, hormone production, and enhancing the immune system, among others (32–34). In this study, we collected data from the largest GWAS to date on gut microbiota and sepsis, and evaluated the causal relationship between all gut microbiota taxa and sepsis. We found that 24 taxa were positively associated with various sepsis outcomes, 30 taxa were negatively associated with sepsis outcomes. In total, we identified 37 unique taxa, including 23 at the genus level, 5 at the family level, 3 at the order level, 4 at the class level, and 2 at the phylum level. After multiple-testing correction, phylum Lentisphaerae, class Lentisphaeria, and order Victivallales were still associated with a reduced risk of sepsis, while Phylum Tenericutes and class Mollicutes were linked to an increased risk of sepsis (particularly in individuals under 75 years old) and 28-day mortality. Notably, we did not observe any significant association between sepsis and these gut microbiota. Taken together, our findings provide valuable insights into the role of gut microbiota in sepsis treatment, including reducing the risk of sepsis, minimizing mortality, and improving sepsis prognosis.

Tenericutes and Mollicutes are primarily associated with infections in pregnant women and newborns. Several studies have shown that mycoplasma infections can cause puerperal sepsis (35), and in newborns, these infections are linked to an increased risk of bronchopulmonary dysplasia, early-onset neonatal sepsis, and meningitis (36, 37). In contrast, Lentisphaerae (phylum), Lentisphaeria (class), and Victivallales (order) are relatively under-studied bacterial groups. However, recent research suggests that these microbial communities are closely associated with immune regulation. Lentisphaerae, for instance, has been found to be more abundant in cases of inflammatory bowel diseases (38), while its abundance is reduced in patients with rosacea (39). Furthermore, in patients diagnosed with post-traumatic stress disorder, Lentisphaerae has been associated with a decrease in symptom severity scores (40). genus Gordonibacter is primarily found to be excessively increased in patients with Crohn’s disease and Rheumatoid Arthritis (RA), which indicates its close relationship with immunity and inflammation (41). This also indirectly confirms its association with the increase in CRP.

Previous MR analyses have suggested that gut microbiota and their metabolites can impact Systemic Lupus Erythematosus, inflammatory bowel diseases, and blood metabolites (42–44). These findings emphasize the significance of these bacterial groups in regulating inflammation in the human body. Their presence and abundance in various disease conditions imply a potential role in modulating immune responses and contributing to the development or resolution of inflammation-related disorders.

Recently, a study employed regression analysis to investigate the potential impact of the interaction between gut microbiota and CRP using individual level genotype data from UK Biobank (45). Nonetheless, due to the insufficient research on the relationship between gut microbiota and serum inflammation, we examined the effect of CRP, an inflammation protein linked to a higher risk of infections in adults (15), in the association between gut microbiota and sepsis. Our findings indicate that Phylum Tenericutes and class Mollicutes are strongly associated with increasing levels of C-reactive protein. Previous studies have shown elevated levels of CRP in patients with mycoplasma infection. Taken together with our results, this implies that CRP might not only work as a biomarker for mycoplasma infection but also play a role in mediating the pathogenic mechanisms of mycoplasma. These results establish the role of certain gut microbiota in systemic inflammation and immune response.

Current research on the effects of serum substances on sepsis has primarily focused on lipid and iron metabolism (46, 47). Several cross-sectional studies have demonstrated that elevated CRP levels are linked to increased morbidity and mortality in sepsis (48–50). In our examination of the relationship between CRP and sepsis, we found that CRP is associated with a higher incidence of sepsis and sepsis-related deaths among those under 75 years of age. Reverse analysis revealed no effect on CRP. Meanwhile, mediation analysis found that CRP mediates 32% of the effects of Phylum Tenericutes and class Mollicutes on sepsis (under 75 years). Based we used multivariate MR, the effect of CRP on sepsis were independent of the effect of the exposure (17). Our Mendelian randomization study on the relationship between our microbiota and the risk of developing and dying from sepsis will help us understand how changes in the gut microbiome lead to immune dysregulation in sepsis, which in turn can aid in improving sepsis management.

Firstly, our study used multiple sensitive analysis, thereby bolstering the reliability of our findings. The consistency between the most of the WM and MR-Egger methods with those from the IVW method attests to the robustness of our results. Despite the presence of wide confidence intervals in some results, the overarching pattern of associations remained consistent. Secondly, we implemented the MR-PRESSO technique to identify and exclude potential outliers that could introduce bias into our findings, enhancing the reliability of our results. Thirdly, our study was instrumental in spotlighting certain genera that showed a more significant association with sepsis compared to other microbial classes. Even though these associations didn’t retain their statistical significance after multiple testing adjustment, they still constitute crucial preliminary observations and may be indicative of underlying biological phenomena. Fourthly, through the use of PhenomeScan, we found that no SNPs from the microbiota, CRP and sepsis were associated with infections, malignant diseases, or antibiotic use. This suggests that the observed links among the microbiota, CRP, and sepsis were unlikely to be confounded by the genetic predispositions that are typically represented by SNPs. Lastly, given that both the exposure and outcome populations were of European descent, the potential for bias resulting from population stratification was minimized.

However, there are several limitations to our study. Firstly, a limited amount of non-European population data on gut microbiota was obtained, which may have biased our findings. Secondly, we were unable to discern any non-linear correlations among mcirbiota, CRP and sepsis, such as U-shaped, J shaped patterns. Thirdly, the number of loci related to CRP is relatively small compared to those associated with sepsis and gut microbiota. Fourthly, our Mendelian randomization study was unable to access individual-level data, which posed a constraint on the depth of our analysis. For instance, we were unable to perform a hierarchical analysis, specifically in the case of sepsis. Ideally, we would have liked to divide the sepsis data into two groups according to the Sepsis-2 and Sepsis-3 guidelines, which could provide insights into the differences between these two classifications. However, due to the unavailability of the required individual-level data, we were unable to conduct such an analysis.

## 5 Conclusion

In conclusion, our bi-directional Mendelian randomization analysis has clearly indicated a causal relationship between the 37 unique gut microbiota taxa and increased risk of sepsis, whereas the reverse causality hypothesis did not hold. Importantly, our findings suggest that C-reactive protein (CRP) acts as a mediator of the impact of the gut microbiota on sepsis. For a more nuanced understanding of the observed association between the gut microbiota and sepsis, future research should focus on potential mechanistic pathways, while also attempting to adjust for potential confounders such as diet, lifestyle, and medication, provided these data are available. Furthermore, an analysis of sepsis as a heterogeneous condition, acknowledging its multi-stages and variations as defined by the sepsis-3 criteria, would be beneficial, throught acquire individual-level data in future. Our work constitutes a significant stride in deciphering the relationship between gut microbiota and sepsis, however, more experimental and clinical studies are warranted to verify and extend our findings. It is our hope that our study acts as a catalyst for further exploration in this field, and thereby contribute to the ceaseless enhancement of patient care in intensive care units.

## Data availability statement

The original contributions presented in the study are included in the article/**Supplementary Material**. Further inquiries can be directed to the corresponding author.

## Author contributions

ZZ, LC, and DN designed the study and collected and collated the data. DN analyzed the data, LC and ZZ drafted the paper. All authors contributed to the article and approved the submitted version.
